# Detection of Bacteriophage Particles Containing Antibiotic Resistance Genes in the Sputum of Cystic Fibrosis Patients

**DOI:** 10.3389/fmicb.2018.00856

**Published:** 2018-05-01

**Authors:** Maryury Brown-Jaque, Lirain Rodriguez Oyarzun, Thais Cornejo-Sánchez, Maria T. Martín-Gómez, Silvia Gartner, Javier de Gracia, Sandra Rovira, Antonio Alvarez, Joan Jofre, Juan J. González-López, Maite Muniesa

**Affiliations:** ^1^Department of Genetics, Microbiology and Statistics, Faculty of Biology, University of Barcelona, Barcelona, Spain; ^2^Department of Clinical Microbiology, Hospital Vall d’Hebron, Vall d’Hebron Institut de Recerca (VHIR), Universitat Autònoma de Barcelona, Barcelona, Spain; ^3^Cystic Fibrosis Unit, Hospital Universitario Vall d’Hebron, Universitat Autònoma de Barcelona, CIBER of Respiratory Diseases (Ciberes CB06/06/0030), Carlos III Health Institute, Barcelona, Spain

**Keywords:** cystic fibrosis, bacteriophages, antibiotic resistance genes, horizontal gene transfer, sputum

## Abstract

Cystic fibrosis (CF) is a chronic disease in which the bacterial colonization of the lung is linked to an excessive inflammatory response that leads to respiratory failure. The microbiology of CF is complex. *Staphylococcus aureus* is the first bacterium to colonize the lungs in 30% of pediatric CF patients, and 80% of adult patients develop a chronic *Pseudomonas aeruginosa* infection, but other microorganisms can also be found. The use of antibiotics is essential to treat the disease, but antibiotic performance is compromised by resistance mechanisms. Among various mechanisms of transfer of antibiotic resistance genes (ARGs), the recently been reported bacteriophages are the least explored in clinical settings. To determine the role of phages in CF as mobile genetic elements (MGEs) carrying ARGs, we evaluated their presence in 71 CF patients. 71 sputum samples taken from these patients were screened for eight ARGs (*bla*_TEM_, *bla*_CTX-M-1_-group, *bla*_CTX-M-9_-group, *bla*_OXA-48_, *bla*_VIM_, *mecA*, *qnrA*, and *qnrS*) in the bacteriophage DNA fraction. The phages found were also purified and observed by electron microscopy. 32.4% of CF patients harbored ARGs in phage DNA. β-lactamase genes, particularly *bla*_VIM_ and *bla*_TEM_, were the most prevalent and abundant, whereas *mecA*, *qnrA*, and *qnrS* were very rare. *Siphoviridae* phage particles capable of infecting *P. aeruginosa* and *Klebsiella pneumoniae* were detected in CF sputum. Phage particles harboring ARGs were found to be abundant in the lungs of both CF patients and healthy individuals and could contribute to the colonization of multiresistant strains.

## Introduction

Cystic fibrosis (CF) is the most common autosomal recessive disease in the Caucasian population (Panitch and Rubenstein, 2010). The important morbidity and mortality of this disease are related to pulmonary affectation and its complications, which are responsible for up to 85% of the deaths of patients suffering from CF, including children and adults (Panitch and Rubenstein, 2010; Elborn, 2016).

The microbiology of CF is complex. *Staphylococcus aureus* colonizes the lungs of more than 30% of child or youth patients. In adolescence, chronic pulmonary infection with *Pseudomonas aeruginosa* is developed, which remains in up to 80% of adult CF patients and is capable of growing in biofilms in the lungs, thereby greatly complicating antibiotic treatment of the disease. Other opportunistic pathogens have also been isolated from CF patients, including *Burkholderia cepacia* and *Haemophilus influenza.* Additional opportunistic pathogens such as *Stenotrophomonas maltophilia, Achromobacter xylosoxidans*, and nontuberculous *Mycobacterium* are being recovered from adult patients with increasing frequency (Bittar et al., 2008; Lipuma, 2010).

After the observation that the mucous obstructing CF airways is hypoxic, analysis under strict anaerobic conditions has revealed that anaerobic bacterial species are also present within CF airways in high numbers. The spectrum of facultative and obligate anaerobic species recovered from CF samples frequently includes members of the genera *Prevotella*, *Streptococcus*, *Rothia*, and *Veillonella* (Fodor et al., 2012). High throughput sequencing efforts indicate that the CF microbiome consists of more than 60 different bacterial genera, while interrogation of bacterial 16S ribosomal RNA (rRNA) gene-based phylogenetic microarrays has placed the estimate at as many as 43 different bacterial phyla and over 2,000 different taxa (Guss et al., 2011; Fodor et al., 2012). In contrast, the role of fungi, viruses and mycobacteria (which are not identified by standard bacterial 16S rRNA sequencing) is still unclear (Kim et al., 2015). Pulmonary exacerbations are related to a complex relationship between host defense and airway microbiota and can lead to pulmonary decline. Early recognition and treatment of exacerbations is fundamental for patient wellbeing, and so frequent monitoring of patients is necessary.

Antibiotic resistance is a serious global health problem (Hawkey and Jones, 2009), as infections caused by resistant pathogens may be difficult to treat. The dispersion of clones exhibiting resistance to various antibiotics has become common (Cantón et al., 2005; Czekalski et al., 2012) and represents a greater threat than ever before (Hawkey, 2008; Hawkey and Jones, 2009). In the case of CF, the use of antimicrobials is necessary throughout the life of the patient. Aggressive antibiotic therapies are already used during primocolonization with the aim of eradicating the microorganisms in the airway of the CF patient. The ultimate objective is to delay the deterioration of lung function that occurs during chronic colonization, which reduces the quality of life and survival of the patient (Castellani and Assael, 2017). During chronic colonization, nebulized, oral, and intravenous chronic suppressive treatments are used (Cantón et al., 2005; Castellani and Assael, 2017). All this facilitates resistant or multiresistant antimicrobial bacteria being selected over time. Some studies suggest that up to 45% of CF patients are colonized by a multiresistant microorganism, which makes antimicrobial treatment even more difficult (McCaughey et al., 2013). Among new proposals for treating CF infections, phage therapy and co-treatment with antibiotics is a promising approach that may overcome antibiotic resistant pathogens and natural resistance in biofilm (Alemayehu et al., 2012; Saussereau et al., 2014; Fong et al., 2017).

Transfer of antibiotic resistance genes (ARGs) mediated by mobile genetic elements (MGEs) provides the most important and rapid mechanism of dispersion. The most commonly studied MGEs are plasmids, transposons and, more recently, bacteriophages (or phages) (Srivastava et al., 2004; Colomer-Lluch et al., 2011b; Colombo et al., 2016; Lekunberri et al., 2017), which are bacterial viruses that infect and multiply using the machinery of the host bacterium.

In the case of CF, phages have been detected in the metagenome of respiratory tracts of patients (Willner et al., 2009) and ARGs have been found in the sputum virome of five patients (Fancello et al., 2011). Phages can be important contributors to the mobilization of ARGs, leading to the emergence of new resistant clones, which is a major problem in the treatment of CF patients. Our objective was to detect and quantify phage particles carrying ARGs in sputum samples of CF patients.

## Materials and Methods

### Samples

The study was conducted with 71 sputum samples of 71 CF patients taken during regular follow up visit to the CF unit of Vall d’Hebron Hospital in Barcelona, from August 2015 to October 2016. The age of CF patients under study ranged from four to 79 years. All the samples were used only after performing a conventional microbiological diagnosis and they were completely anonymized. No data other than the age of the patients were collected and the samples were destroyed immediately after the study. The study was approved by the Clinical Research Ethics Committee of the Hospital (reference number PR(AG)187/2014). Additionally, 21 sputum samples were taken from a group of people not suffering from CF who had not received antibiotics in the previous 3 months.

### Bacterial Isolation and Antimicrobial Susceptibility Characterization

Sputum samples were processed within 2 h of reception. They were homogenized with 2% cysteine solution (1:1) (v:v) and vigorously vortexed before being plated in chocolate agar, mannitol salt agar, MacConkey agar, and modified Thayer-Martin agar.

Isolates identification was performed using the VITEK MS matrix-assisted laser desorption/ionization time-of-flight mass spectrometry (MALDI-TOF MS) system (bioMérieux, Marcy-l’Étoile, France) and antimicrobial susceptibility to β-lactams, quinolones and aminoglycosides of bacterial isolates was studied by the disk diffusion method following EUCAST (EUCAST, 2016). Specifically, the antimicrobials evaluated for non-fermenting gram-negative bacteria were ampicillin-sulbactam, piperacillin-tazobactam, ceftazidime, cefepime, aztreonam, imipenem, meropenem, ciprofloxacin, amikacin, and tobramycin; and for *Staphylococcus aureus*, penicillin, ampicillin, cefoxitin and gentamicin.

### Bacteriophage Purification From Sputum Samples

One ml of each sample was diluted 1:3 in phosphate-buffered saline (PBS). The suspension was filtered through 0.22 μm low protein-binding membrane filters (Millex-GP, Millipore, Bedford, MA, United States). The suspension was then treated with chloroform (1:10) (v:v) to minimize the presence of membrane vesicles containing DNA and treated with DNase (100 units/ml of the phage lysate at 37°C for 1 h), to remove non-packaged DNA. The DNase was heat inactivated at 75°C for 5 min.

### Evaluation of the Protocol for Phage DNA Recovery From Sputum Samples

Assays were performed to verify the efficiency of the protocol for phage DNA recovery from sputum. Firstly, various degrees of homogenization of five CF sputum samples negative for the presence of the *bla*_TEM_ gene were performed in PBS (direct analysis, 1:3 dilution and 1:5). In each dilution, 10 μl of the qPCR standard containing 10^3^ gene copies (GC) of the *bla*_TEM_ gene were inoculated. The conditions showing the best recovery of the GC number were used in the experiments.

In addition, five CF sputum samples were inoculated to a final concentration of 10^6^ phage particles/ml with a suspension of phage 933W (Imamovic et al., 2010). This phage contains one copy of the Shiga toxin 2 (*stx*_2_) gene in its genome and was not expected to be naturally present in these samples. The *stx*_2_ qPCR assay (Imamovic et al., 2010; **Table 1**) was used to detect the *stx* and each copy corresponded to one phage 933W. Comparison of the number of phage particles inoculated and the number of phages 933W recovered from the sputum after phage purification and DNA extraction allowed the protocol effectiveness to be calculated.

**Table 1:** Quantitative real time PCR primers and probes for qPCR assays used in this study.

Target gene	PCR	Sequence	Amplimer (bp)	Reference
*bla*_TEM_ qPCR	UP	CACTATTCTCAGAATGACTTGGT	85	Lachmayr et al., 2009
	LP	TGCATAATTCTCTTACTGTCATG		
	Probe	6FAM-CCAGTCACAGAAAAGCATCTTACGG-MGBNFQ		
*bla*_CTX-M-1_ qPCR	UP	ACCAACGATATCGCGGTGAT	101	Colomer-Lluch et al., 2011b
	LP	ACATCGCGACGGCTTTCT		
	Probe	6FAM – TCGTGCGCCGCTG-MGBNFQ		
*bla*_CTX-M-9_ qPCR	UP	ACCAATGATATTGCGGTGAT	85	Colomer-Lluch et al., 2011a
	LP	CTGCGTTCTGTTGCGGCT		
	Probe	6FAM – TCGTGCGCCGCTG-MGBNFQ		
*bla*_OXA-48_ qPCR	UP	CGGTAGCAAAGGAATGGCAA	133	Brown-Jaque et al., 2018
	LP	TGGTTCGCCCGTTTAAGATT		
	Probe	6FAM – CGTAGTTGTGCTCTGGA-MGBNFQ		
*bla*_VIM_ qPCR	UP	AATGGTCTCATTGTCCGTGATG	61	This study
	LP	TACAGCGTGGGGTGCGA		
	Probe	6FAM –TGATGAGTTGCTTTTGATTG-MGBNFQ		
*mecA* qPCR	UP	CGCAACGTTCAATTTAATTTTGTTAA	92	Volkmann et al., 2004
	LP	TGGTCTTTCTGCATTCCTGGA		
	Probe	6FAM-AATGACGCTATGATCCCAATCTAACTTCCACA-MGBNFQ		
*qnrA* qPCR	UP	AGGATTGCAGTTTCATTGAAAGC	138	Colomer-Lluch et al., 2014b
	LP	TGAACTCTATGCCAAAGCAGTTG		
	Probe	6FAM-TATGCCGATCTGCGCGA-MGBNFQ		
*qnrS* qPCR	UP	CGACGTGCTAACTTGCGTGA	118	Colomer-Lluch et al., 2014b
	LP	GGCATTGTTGGAAACTTGCA		
	Probe	6FAM –AGTTCATTGAACAGGGTGA-MGBNFQ		
*stx*_2_	UP	ACGGACAGCAGTTATACCACTCT	65	Imamovic et al., 2010
	LP	CTGATTTGCATTCCGGAACGT		
	Probe	FAM-CCAGCGCTGCGACACG-NFQ		
16S rDNA	UP 28f	AGAGTTTGATCCTGGCTCAGA	1503	Weisburg et al., 1991
Eubacteria	LP 1492r	TACGGCTACCTTGTTACGACTT		


**qPCR, quantitative real time PCR; UP, upper primer; LP, lower primer; MGBNFQ, Minor groove binding non-fluorescent quencher; FAM: 6-carboxyfluorescein reporter*.*

### Phage DNA Extraction

The phage suspensions purified from the samples or from the enrichment cultures were then digested using proteinase K (0.5 μg.ml^-1^), and the DNA extracted with phenol/chloroform (1:1 *v*/*v*) (Sambrook and Russell, 2001). The remaining phenol/chloroform was removed by adding the mixture to Phase Lock Gel Tubes (5-Prime, Hucoa Erlöss, Madrid, Spain) and centrifuging following the manufacturer’s instructions. DNA was precipitated using 100% ethanol and 3M sodium acetate, and resuspended in 50 μl of ultrapure water. DNA was quantified using a NanoDrop ND-1000 spectrophotometer (NanoDrop Technologies, Thermo Fisher Scientific, Wilmington, DE, United States). To verify the absence of non-packaged DNA, the protocol for DNA extraction from the phage fraction of the samples was always accompanied by several controls, as described previously (Colomer-Lluch et al., 2014a). To rule out the possibility of contamination with free DNA outside the phage particles, an aliquot of the sample taken after DNase treatment and before desencapsidation was evaluated. At this stage, the samples were also used as a template for conventional PCR of eubacterial 16S rDNA (**Table 1**) and as a template for the qPCR assay of each ARG. Both amplifications should be negative, confirming that DNase has removed all non-encapsidated DNA from the samples.

### qPCR Procedures

Eight clinically relevant ARGs that differ in their resistance mechanisms and clinical significance were evaluated: five genes that confer resistance to β-lactam antibiotics (*bla_TEM_*, *bla*_*CTX-M*-1_ group, *bla*_CTX-M-9_ group *bla*_OXA-48_, and *bla*_VIM_), two quinolone resistance genes (*qnrA* and *qnrS*) and a gene conferring resistance to methicillin (*mecA*), commonly found in *Staphylococcus* (Colomer-Lluch et al., 2011b). A fragment of each target ARG was amplified by conventional PCR using an Applied Biosystems 2720 Thermal Cycler (Applied Biosystems, Barcelona, Spain) with the primers described previously (Brown-Jaque et al., 2018), purified and cloned into a pGEM-T Easy vector for insertion of PCR products (Promega, Barcelona, Spain), and used to generate the standard curves as previously described (Colomer-Lluch et al., 2011b). The standard curves were constructed with the averaged values obtained by three replicates in at least five independent serial dilutions of the standard.

TaqMan qPCR assays (**Table 1**) were performed under conditions described previously (Colomer-Lluch et al., 2011b) in a Step One Real-Time PCR System (Applied Biosystems, Spain). Genes were amplified in a 20 μl reaction mixture with the TaqMan^®^ Environmental Master Mix 2.0 (Applied Biosystems). The reaction contained 2 or 9 μl of the sample DNA or quantified plasmid DNA.

All the samples, standards used for quantification of each ARG, positive controls of DNA containing each ARG and negative controls added to rule out the presence of non-encapsidated DNA (Colomer-Lluch et al., 2014a) and contamination of the qPCR reaction, were assayed in duplicate. The gene copy (GC) was defined as the average of the duplicate data obtained. The efficiency (*E*) of all qPCR reactions ranged from 95 to 100%. To quantify the ARGs we considered the GC results obtained within the threshold cycle (Ct) within the limit of quantification (LOQ). This was determined by the last valid Ct for each ARG assay in the standard curve that is consistent in the diverse replicates. However, sometimes amplification was observed beyond the LOQ, but the lack of consistency in the replicates did not allowed the GC to be correctly quantified. The values beyond the LOQ but not undetermined were those within the limit of detection (LOD).

We designed a new *bla*_VIM_ qPCR assay for this study. A 748 bp fragment of the sequence of *bla*_VIM_ was amplified from a clinical isolate of *P. aeruginosa.* by conventional PCR with primers UP-TCTACATGACCGCGTCTGTC/LP-TGTGCTTTGACAACGTTCGC. The fragment generated was cloned in a pGEM vector and used to prepare the standard curve. A *bla*_VIM_ qPCR assay (**Table 1**) was designed with the software tool Primer Express 3.0 and commercially synthesized (Applied Biosystems). Specificity was determined via sequence alignments using *bla_VIM_* sequences available in the NCBI nucleotide database. A FAM-labeled fluorogenic probe was commercially synthesized by Applied Biosystems. The *bla_VIM_* probe was minor-groove binding (MGB) with an FAM reporter (FAM: 6-carboxyfluorescein) and an NFQ quencher (non-fluorescent quencher). The *bla*_VIM_ gene showed an efficiency of 91.9% and a detection limit of 22.5 GC/μl (Ct of 33.9).

### Evaluation of Infectious Phages in the Samples

Laboratory strain *Escherichia coli* WG5 (Anonymous, 2000), *S. aureus* RN450 (Novick, 1967), an environmental isolate of *P. aeruginosa* and *Klebsiella pneumoniae* reference strain of capsular serotype K2 (Orskov and Orskov, 1984), were used as hosts for bacteriophage propagation. These were selected for their ability to detect lytic phages and were negative for the ARGs in this study. Phage propagation was performed in solid culture by double agar layer (Anonymous, 2000) with some modifications. Briefly, 1 ml of target bacteria grown in LB at an OD_600_ of 0.3 was mixed with Luria Bertrani (LB) semi-solid agar (0.7% agar) supplemented with 6 mM CaCl_2_. The mixture was poured into LB agar plates and left to solidify at room temperature. Ten microliters of each phage suspension was dropped onto the agar layer and plates were examined for the presence of lysis after incubation at 37°C overnight.

### Phage Purification by CsCl Density Gradients

Sputum samples showing a high level of ARGs in phage DNA were pooled and used for purification of phages using caesium chloride (CsCl) density gradients (Sambrook and Russell, 2001). The easily visible gray bands corresponding to bacteriophages were collected and dialysed. Phages in the bands were used in electron microscopy studies and to quantify the presence of ARGs by qPCR.

### Electron Microscopy Studies

Fifteen microliters of phage suspensions purified from CsCl gradient sputum was deposited on copper grids with carbon-coated Formvar films and negatively stained with 2% ammonium molybdate (pH 6.8) for 1.5 min. The samples were examined in a Jeol 1010 transmission electron microscope (JEOL USA Inc., Peabody, MA, United States) operating at 80 kV.

### Statistical Analysis

Analysis was carried out using the R software packages (R Core Team, 2014). An ANOVA test was used and a 5% significance level was adopted to identify differences in ARG content in phage DNA between groups of individuals under or over 18 years of age.

## Results

### Evaluation of the Protocol for Phage DNA Recovery From Sputum Samples

Homogenization of the sputum sample in PBS (1:3) (v:v) proved to be equally as efficient as homogenization in PBS (1:5) (v:v). The preliminary assays performed to evaluate the recovery of spiked *bla*_TEM_ GC showed a recovery of >99% with respect to the stock. In order to minimize the dilution of the sample, 1:3 was selected for the study.

Recovery of Stx phage 933W spiked in the samples ranged from 96.0 to 99.8% in five CF sputum samples, suggesting that the purification protocol efficiently recovered viral particles inoculated in the samples.

### Occurrence of ARGs in Phage DNA of CF Sputum Samples: Limit of Detection Versus Limit of Quantification for Positive Samples

The LOD is the absolute highest threshold cycle (Ct) generated that still proves to be amplification of the specific target sequence. The LOQ is defined as the last valid Ct in the standard curve that is consistent in the diverse replicates and is used to calculate the efficiency of each qPCR assay. In our case, *C*t-values above 34–35 were close to the borderline of true detection and produced inconsistent and non-reproducible results among the replicates due to the low GC numbers.

**FIGURE 1:**
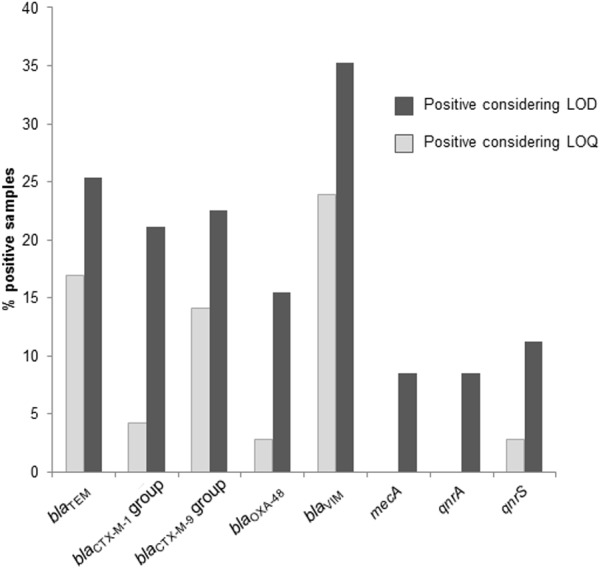
Percentage of positive samples for each antibiotic resistance gene (ARG) in phage DNA of the sputum samples considering the limit of detection (LOD) or the limit of quantification (LOQ) of the qPCR assays.

The results for the different ARGs in the phage fraction of the sputum samples varied according to whether the LOD or LOQ was considered (**Figure 1**). Positive samples within the LOD showed regular curves but the *C*t-values were too close to the limit and beyond the LOQ defined for our qPCR assays. As it was impossible to properly calculate the densities of GC in these cases, only positive samples within the LOQ are included in the results. However, we consider it worth noting that some amplification of certain ARGs took place, which emerge when considering the LOD, and that these samples were not really negative. This is the case of *mecA* and *qnrA* genes. When considering the LOD, some samples carried up to seven ARGs, whereas when considering the LOQ, no more than four ARGs were encountered in the same sample.

### Occurrence of ARG by Age

**FIGURE 2:**
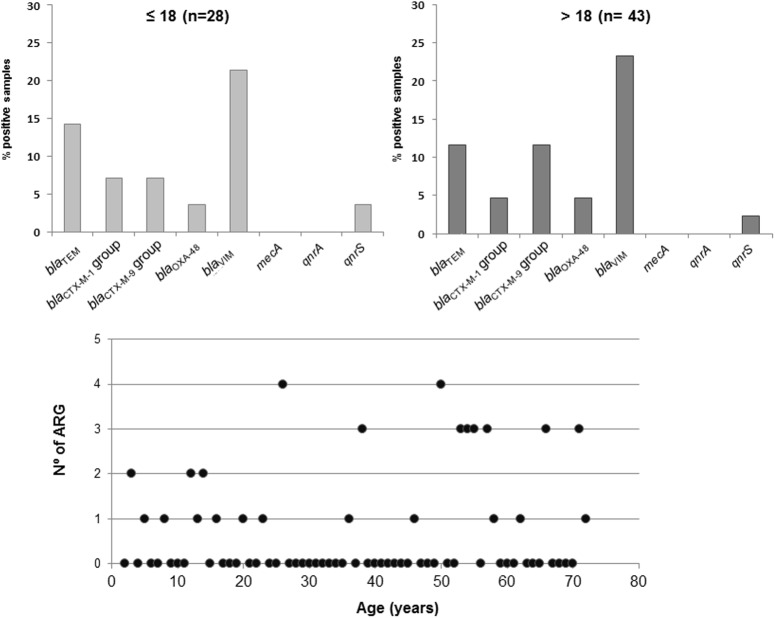
Percentage of positive samples for each ARG in phage DNA of the sputum samples of individuals aged below 18 years and above 18 years old. Lower chart shows the distribution of the number of ARGs in phage DNA detected in each individual in relation to age.

Sputum samples were taken from 71 CF patients aged 4 to 79 years. Two subgroups were devised: patients under or 18 years old (*n* = 28) and above (*n* = 43) (**Figure 2**). No remarkable differences were observed; the only significant (*p* < 0.05) differences were a higher percentage of *bla*_TEM_ and lower percentage of *bla*_CTX-M-9_ group positive samples in the younger subgroup (**Figure 2**).

In addition to the 71 sputum samples from CF patients, sputum samples from 21 healthy individuals not affected by the disease were analyzed. ARGs in the phage fraction were also detected in this group. As for CF samples, *mecA* was not detected in non-CF individuals. One sample was positive for *bla*_CTX-M-1_, *bla*_OXA-48_ and *qnrS. qnrA* and *bla*_CTX-M-9_ were detected in three and four control samples, respectively, and *bla*_TEM_ and *bla*_VIM_ were the most prevalent with seven and eight positive samples, respectively.

### Abundance of ARGs in Phage DNA of Sputum of CF Patients

**FIGURE 3:**
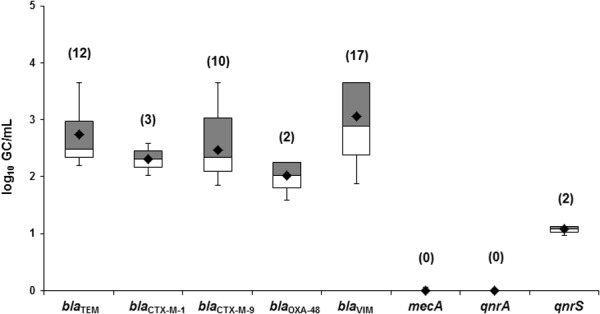
Quantification of ARGs in the phage fraction of sputum samples from cystic fibrosis patients. Values in brackets indicate the number of positive samples that were used for the calculation. In the box plot, the cross-pieces of each box represent (from Top to Bottom) the maximum, upper-quartile, median (black bar), lower-quartile and minimum values. The black diamond shows the mean value. The upper gray boxes in the box plot include samples showing values within the 75th percentile and lower white box samples show values within the 25th percentile.

The quantification of ARGs in phage DNA showed that β-lactamase genes were the most abundant in CF patients, with values of up to 10^3^ GC/ml (**Figure 3**). In contrast, there was an absence of *qnrA* and *mecA*, and very low numbers of *qnrS*. The highest copy number was obtained for the *bla*_VIM_ gene with a mean of 3.1 log_10_ GC/ml (**Figure 3**) and a maximum value of 4.6 log_10_ GC/ml (**Figure 3**).

### Characterization of Bacterial Isolates and Antibiotic Resistances

The results of the bacterial cultures of the sputum samples of the CF patients showed that 46.5% were positive for *P. aeruginosa*, 39.4% for *S. aureus*, 7.0% for *Achromobacter* sp., 5.6% for *Stenotrophomonas maltophilia*, 2.9% for *H. influenzae*, 1.4% for *H. parainfluezae* and 1.4% for *Burkholderia* sp. None of the samples were positive for any species of the *Enterobacteriaceae* family. Antimicrobial susceptibility testing showed that 42.4% of the *P. aeruginosa* isolates obtained were multidrug-resistant (i.e., non-susceptible to at least one agent in three or more antimicrobial categories) and of those, 71.4% were extensively drug-resistant (i.e., susceptible to only one or two antimicrobial categories). Regarding the *S. aureus* isolates obtained, 34.6% of them were methicillin resistant.

In the non-CF group, 14 of the 21 sputum samples were analyzed for the presence of bacteria. In five samples *S. aureus* was detected (one of them was methicillin resistant) and in one sample *P. aeruginosa* was detected. No bacteria were isolated from the rest of the samples.

When comparing the results of isolates for each sample and the ARG results in phage DNA, no co-occurrence was found between ARG in phage DNA and the bacterial species isolated or their antibiotic susceptibility profiles.

### Presence of Infectious Bacteriophages in the CsCl Purified Samples

The qPCR detection of ARGs in the phage DNA fraction of sputum samples provided evidence of the presence of ARGs-mobilizing phage particles. To gain more information, we further purified the phage particles in the samples to (i) evaluate their infectivity, (ii) directly visualize them by electron microscopy, and (iii) confirm that these purified particles propagated in a host strain and observed by electron microscope carried ARGs.

**FIGURE 4:**
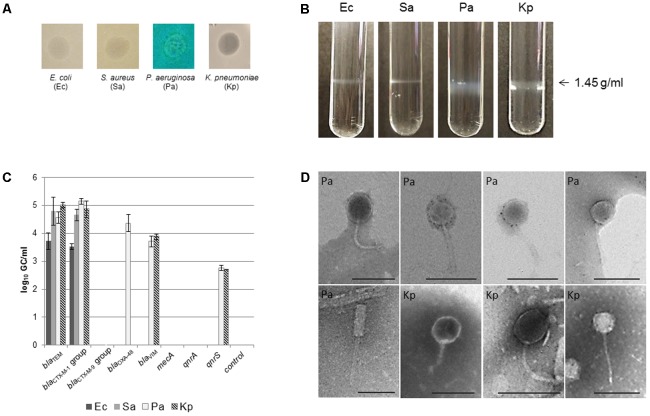
Analysis of infectious phage particles isolated from pooled sputum samples of CF patients. **(A)** Infectivity of the phages from sputum samples onto *Escherichia coli* (Ec), *Staphylococcus aureus* (Sa), *P. aeruginosa* (Pa), and *K. pneumoniae* (Kp) hosts. Gray bands corresponding to density 1.45 g/ml in the tubes of CsCl density gradients prepared with the phages isolated from the spot test onto each strain **(C)** qPCR results of the ARGs present in the phage particles purified from each CsCl density band in **(B)**. **(D)** Electron micrographs of phage particles purified from the CsCl bands obtained with Pa and Kp samples. Bar 100 nm.

A pool of phages purified from 10 sputum samples was used to determine the presence of infectious phages carrying ARGs by a spot test, as described in section “Materials and Methods.” The occurrence of lysis was attributed to the presence of phages capable of infecting a given strain. Opaque lysis in the area of the spot test was observed on the *E. coli* WG5 host strain (Ec) (Brown-Jaque et al., 2016) and *S. aureus* RN450 (Sa) (Novick, 1967), whereas clear lysis occurred on *P. aeruginosa* (Pa) and *K. pneumoniae* (Kp) hosts (**Figure 4A**).

To increase the volume of phage suspension, 20 spots of each strain were pooled together. As a negative control, layers of each host strain without phages were used. Suspensions were treated by chloroform, DNAse and further purified by CsCl gradients.

Thick gray bands at the densities expected for phages (1.45 g/ml) were obtained for phage suspensions recovered from Kp and Pa lysis areas. Thin bands at the same density were obtained from Ec and Sa samples (**Figure 4B**). No bands were obtained from the control without phages. DNAs extracted from phages in each of the bands in **Figure 4B** were used as a template for the qPCR quantification of ARGs (**Figure 4C**). *bla*_TEM_, *bla*_CTX-M-1_ group, *bla*_OXA-48_, and *bla*_VIM_ were detected in densities of up to 10^5^ GC/ml depending on the gene and the host strain (**Figure 4C**).

Particles compatible with phage capsid heads were visualized by electron microscopy in the CsCl bands of Ec and Sa samples, but the presence of phages could not be confirmed due to the absence of tails (images not shown). In contrast, capsids of phages of the *Siphoviridae* morphological types with isometric heads and various tail lengths (120–150 nm) were observed in Pa and Kp samples (**Figure 4D**). We also observed one apparently detached *Myoviridae* tail (210 nm) (**Figure 4D**, Pa, second row).

## Discussion

Phages are among the most recently studied MGEs that play a role in the spread of ARGs (Colomer-Lluch et al., 2011b; Fancello et al., 2011; Subirats et al., 2016). In our study, ARGs were detected in phage particles, as previously observed in other biomes (Muniesa et al., 2004; Colomer-Lluch et al., 2011b; Lekunberri et al., 2017; Lood et al., 2017; Brown-Jaque et al., 2018) and in accordance with metagenomics analyses of CF patients (Bittar et al., 2008; Fancello et al., 2011). The protocol used appeared to be sufficiently efficient for our purposes. However, it should be noted, that CF sputum samples are very dense matrices and the protocol of inoculation and mixture of phage 933W may not have achieved a level of phage particle internalization comparable with that of phage particles already present in the sample. As a consequence, recovery of naturally occurring phages, or phage types other than 933W, could be less efficient than in the spiked samples. In this case, the phage densities in the real samples would be even higher than our results indicate. Furthermore, the limits imposed by our qPCR quantification curve may underestimate the presence of ARGs in some samples, as revealed when comparing the LOD and LOQ.

Compared to healthy individuals, the far greater viscosity of the sputum of CF patients could also hinder the extraction of the packaged DNA, causing an underestimation of the results. It is also possible that the microorganisms remain more firmly attached to the lung in CF patients because of the difficulties in expectoration.

Among all ARGs amplified in this study, the most prevalent and abundant group are the β-lactamase genes. This is in agreement with the widespread expansion of *bla* genes in the environment, either in bacterial (Mesa et al., 2006) or phage fractions (Colomer-Lluch et al., 2011a,b; Marti et al., 2014), in human (Fancello et al., 2011) and animal biomes (Mesa et al., 2006; Economou and Gousia, 2015). Notably, a high percentage of *P. aeruginosa* isolates from CF patients in our study displayed resistance to β-lactam antibiotics, in agreement with previous reports (López-Causapé et al., 2017). A high β-lactamase activity has also been reported in CF-patients (Giwercman et al., 1992). In contrast, the *mecA* gene was almost absent in the phage DNA fraction of our study, while 35% of the isolates were MRSA, in agreement with other studies (Muhlebach, 2017).

Analysis of a smaller group of samples from non-CF-patients also revealed the presence of ARGs, confirming an individual variability of the microbiome (Hauser et al., 2014). Moreover, the healthy population has for decades been subjected to antibiotic pressure and has incorporated ARGs into their microbiomes, even in the absence of a recent antibiotic treatment (Sommer et al., 2010; Penders et al., 2013; Quirós et al., 2014; Brown-Jaque et al., 2018).

An ARG encoded in a bacterial cell can be mobilized by a phage particle through transduction, or a related mechanism not yet defined (Quirós et al., 2016). The particle may remain in a biome where bacteria are no longer present or be mobilized to a different area. This might explain the apparent lack of coherence of our results concerning ARGs in packaged DNA and the bacterial species isolated. We recently demonstrated that phages present in many human biomes can interfere in the isolation of bacteria from samples (Brown-Jaque et al., 2016). This interference could be due to phage infection, propagation and subsequent lysis of the bacteria targeted for isolation during the enrichment process. In addition, only a fragment of the ARG may be detected by qPCR amplification, or the phage particle might not be carrying the complete gene (Martínez et al., 2014) and hence be unable to confer resistance. Nevertheless, in previous studies at least a fraction of ARGs in phage DNA were complete and able to confer resistance (Colomer-Lluch et al., 2011b).

Previous evidence from phage fecal environments or studies of clinical strains suggests that the mobilization of phage-based ARGs may be mostly by generalized transduction (Monson et al., 2011). It has been proposed that the frequency of transduction events could be greater than previously thought (McDaniel et al., 2010). To the best of our knowledge, the phage particles observed after propagation on *P. aeruginosa* and *K. pneumoniae* allowed us to visualize phages in sputum samples for the first time.

The high levels of ARGs in the purified infectious phage particles confirmed that at least some of them harbored ARGs. The number of phage particles present in Ec and Sa samples was apparently insufficient to allow their visualization by electron microscope. Considering that the concentration required for phage detection by electron microscopy is a minimum of 10^8^ particles/ml (Brown-Jaque et al., 2016), it can be estimated that a fraction of 1/10^3^–1/10^5^ of the particles observed in Pa and Kp contained one ARG, a frequency in agreement with previous studies (McDaniel et al., 2010). The ability of some bacteriophages to infect different bacteria, strains or even bacterial genera would facilitate the ARG mobilization in biomes that contain multiple microorganisms such as the lungs.

One of the most important pathogen is *Pseudomonas spp.* (Elborn, 2016). A notably high prevalence of *bla*_VIM_ has been found in packaged DNA, an ARG linked to *Pseudomonas* infection (Tato et al., 2010). *Pseudomonas* cells carry a high number of prophages and generalized transduction has been extensively reported in this genus (Fothergill et al., 2011; Monson et al., 2011). Phages of *Pseudomonas* appear to be polyvalent and can even infect other bacterial genera (for instance *E. coli*) (Yu et al., 2016). In the early stages of the disease, the lungs are colonized by *S. aureus* (Bittar et al., 2008; Elborn, 2016), which displays mobilization of pathogenicity islands (encoding virulence factors) through helper phages (Novick et al., 2010) and in *S. aureus* phages capable of mobilizing ARGs have also been reported (Novick et al., 2010). Moreover, *Burkholderia*, another common lung colonizer in CF patients, is the first non-α proteobacteria reported to contain gene transfer agents (GTAs), phage-derived elements encoded in the bacterial chromosome capable of packaging any sort of bacterial DNA in their phage capsids (Ronning et al., 2010). GTAs involve a mechanism similar to generalized transduction (Ronning et al., 2010; Quirós et al., 2016), in which the DNA of the bacteria carrying the GTA genes is packaged and spread within the capsids they encode. Phages and phage-like elements are also common in pathogens associated with CF, such as *Haemophilus* (Zehr et al., 2012) or *Mycobacterium* (Dedrick et al., 2017).

Most bacteria involved in CF (e.g., *Pseudomonas*), are organized, spread and coexist within the biofilm generated in the lungs of sufferers, which greatly hampers antibiotic treatments. This is about a cause of concern, considering that bacteriophages might transfer genes within biofilms (Solheim et al., 2013).

Transduction rates are highly dependent on the density of bacterial cells and phage particles, and could be increased by various factors: the immobilization of the donor and receptor bacteria in the biofilm matrix, the high concentration of microorganisms in this habitat, and phage induction by antibiotics, which increases the number of phage particles (Fothergill et al., 2011). This could represent another difficulty for the treatment of CF patients, in addition to the antibiotic diffusion barrier of biofilm.

## Ethics Statement

Clinical Ethics Committee approved this study [reference number PR(AG)187/2014]. The laboratories involved in this study are allowed to work with microorganisms classified within biosafety levels 2 and 3.

## Author Contributions

MM, SG, JG, and JG-L designed the study, analyzed and interpreted the results. MM, JJ, and JG-L wrote the draft manuscript. MB-J, LRO, TC-S, and MM-G performed the experiments. JG-L, SG, JG, SR, and AA collected the samples and provided the isolates for this study. MM and JG-L coordinated the study. All authors revised and approved the final version of the manuscript.

## Conflict of Interest Statement

The authors declare that the research was conducted in the absence of any commercial or financial relationships that could be construed as a potential conflict of interest.
